# Improving the Thermostability of a Fungal GH11 Xylanase via Fusion of a Submodule (C2) from Hyperthermophilic CBM9_1-2

**DOI:** 10.3390/ijms23010463

**Published:** 2021-12-31

**Authors:** Huabiao Miao, Yu Ma, Yuanyuan Zhe, Xianghua Tang, Qian Wu, Zunxi Huang, Nanyu Han

**Affiliations:** 1 Engineering Research Center of Sustainable Development and Utilization of Biomass Energy, Ministry of Education, Kunming 650500, China; ynsfmhb@user.ynnu.edu.cn (H.M.); tangxianghua@ynnu.edu.cn (X.T.); wuqian@ynnu.edu.cn (Q.W.); 2 School of Life Science, Yunnan Normal University, Kunming 650500, China; vacavillexiaoyu@gmail.com (Y.M.); lenin1995@user.ynnu.edu.cn (Y.Z.)

**Keywords:** GH11 xylanase, CBM9_1-2, fusion enzyme, thermostability

## Abstract

Xylanases have been applied in many industrial fields. To improve the activity and thermostability of the xylanase CDBFV from *Neocallimastix patriciarum* (GenBank accession no. KP691331), submodule C2 from hyperthermophilic CBM9_1-2 was inserted into the N- and/or C-terminal regions of the CDBFV protein (producing C2-CDBFV, CDBFV-C2, and C2-CDBFV-C2) by genetic engineering. CDBFV and the hybrid proteins were successfully expressed in *Escherichia coli* BL21 (DE3). Enzymatic property analysis indicates that the C2 submodule had a significant effect on enhancing the thermostability of the CDBFV. At the optimal temperature (60.0 °C), the half-lives of the three chimeras C2-CDBFV, CDBFV-C2, and C2-CDBFV-C2 are 1.5 times (37.5 min), 4.9 times (122.2 min), and 3.8 times (93.1 min) longer than that of wild-type CDBFV (24.8 min), respectively. More importantly, structural analysis and molecular dynamics (MD) simulation revealed that the improved thermal stability of the chimera CDBFV-C2 was on account of the formation of four relatively stable additional hydrogen bonds (S42-S462, T59-E277, S41-K463, and S44-G371), which increased the protein structure’s stability. The thermostability characteristics of CDBFV-C2 make it a viable enzyme for industrial applications.

## 1. Introduction

Carbohydrate degradation involves a series of hydrolases, especially xylanase (EC 3.2.1.8) [1]. Xylanase catalyzes the hydrolysis of xylan, a major constituent of hemicelluloses. The enzyme has sparked renewed interest due to its industrial applications, including the paper and pulp sectors as well as the food and feed industries [2]. The application value of xylanases in industrial processes depend on their thermal stability and activity. In some procedures, such as production drying, feed pelleting, maltification, etc., xylanases with excellent thermal stability to adapt high-temperature environments are highly demanded [3]. However, most natural xylanases belong to mesophilic enzymes [4]. Although several xylanases have been extracted from thermophilic microorganisms, their expression levels and enzymatic activities are insufficient for industrial use [5]. Thus, many projects have been undertaken to develop xylanases with improved thermostability and activity to create novel enzymes withstanding harsh conditions [3,6,7,8].

Most xylanases from different species are categorized into glycoside hydrolase (GH) families 10 and 11, with the remainder belonging to families 5, 8, 30, and 43 based on the sequence similarities in the catalytic domain [9]. GH11 xylanases, in comparison to GH10 xylanases, have received significant attention because of their small scale, strict substrate selectivity, high catalytic efficiency, and high temperature and optimal pH ranges. Moreover, most thermophilic xylanases belong to GH11 xylanases [10]. Therefore, developing novel thermotolerant enzymes based on GH11 xylanases has become an intense research direction due to the high temperature requirements in many industrial processes.

Carbohydrate-binding modules (CBMs) are divided into different families based on their sequence similarities, and to date, 88 families are listed in the online database CAZy (http://www.cazy.org, accessed on 10 October 2021) [11]. In addition to participating in substrate binding, some CBMs affect enzyme thermostability. For example, CBM22 from XynA of *Thermotoga maritima* was fused with Xyn2 of *Trichoderma reesei,* resulting in increased thermostability [3]. With the fusion of CBM22 from hyperthermophilic *Thermotoga neapolitana*, the thermostability of *Bacillus halodurans* xylanase was reduced [12]. The addition of CBM3 to the catalytic domain of endoglucanase CelA of *Clostridium thermocellum* did not affect its thermostability [13]. However, removing CBM22 from XynC of *C. thermocellum* decreased its thermostability, but CBM36 deletion from Xyn11 of *Caldicellulosiruptor* sp. F32 improved its thermostability [14,15]. In the current CBM family, 80.0% of them are between 50 and 100 amino acids in length [11]. Relevant studies have shown that larger CBMs do not have an advantage in improving the properties of chimeras [1] and even reduce the thermal stability of wild enzymes [12,16]. This is because a larger CBM usually contains multiple smaller serial submodules inside, and these submodules have different functions [1,16,17]. CBMs or submodules with a shorter length have a simpler protein structure and a clearer influence on the spatial structure of wild enzymes, but they are more popular [1,12,16,17]. Meanwhile, there are reports that by fusing the hyperthermophilic CBM9_1-2 module derived from *T. maritima* GH10 xylanase A to the C-terminus of xylanase from *Aspergillus niger* GH11, the thermal stability of the Xyn-CBM9_1-2 chimera is slightly reduced [1]. However, after subdividing hyperthermophilic CBM9_1-2 into two smaller submodules, the C2 submodule can significantly improve the activity and thermostability of *A. niger* xylanase [16]. Although many studies on the role of CBM have been reported [1,3,12,13,14,15,16], the effect of adding CBM to the N-terminus, C-terminus, or both termini has not been performed on GH11 xylanase (CDBFV), with all advantageous propeties from the ruminal fungus *N. patriciarum* but lacks a CBM in the wild xylanase sequence [18].

The effects of adding the C2 submodule from hyperthermophilic CBM9_1-2, according to the database annotation for *T. maritima* xylanase (Swiss-prot: Q60037), to the N- and C-termini and both termini of the catalytic domain of CDBFV on the activity, thermostability, substrate-binding properties, and kinetic parameters of the variants are described in this study. In addition, the specific influence of the C2 submodule on the structure of CDBFV is explained through structural analysis and molecular dynamics simulation.

## 2. Results

### 2.1. The Fusion Gene and the Chimeric CDBFV Variants

**Figure 1:**
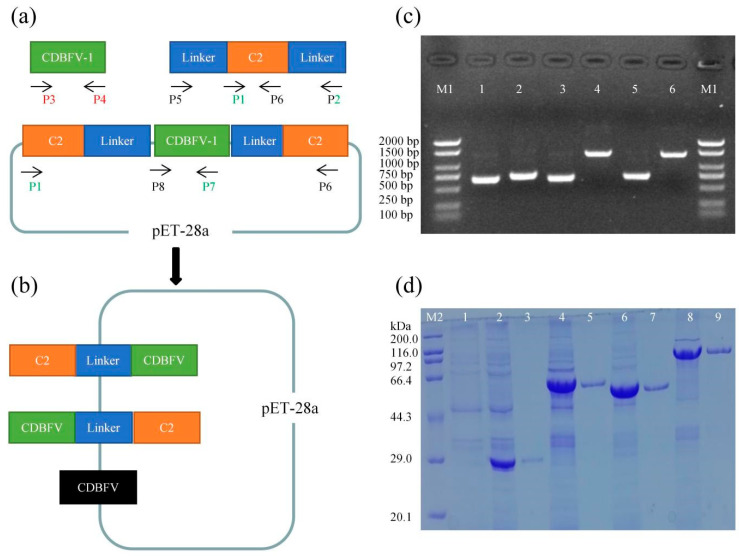
Construction of the chimeras. (**a**) Construction of the C2-CDBFV-C2 chimera model diagram. The C2L, CDBFV-1, and LC2 genes were amplified using P1/P2, P3/P4, and P5/P6, respectively. Orange represents the C2 gene; blue represents the linker gene; green represents the CDBFV gene; the arrow represents the direction of amplification. (**b**) Construction of the CDBFV, C2-CDBFV, and CDBFV-C2 model diagrams. The C2-CDBFV, CDBFV-C2, and CDBFV genes were amplified by using P1/P7, P6/P8, and P7/P8, respectively. (**c**) Agarose gel electrophoresis of amplified fragments. Lane M1: DNA molecular weight marker. Lane 1: gene of CDBFV-1. Lane 2: gene of C2L. Lane 3: gene of LC2. Lane 4: gene of CDBFV-C2. Lane 5: gene of CDBFV. Lane 6: gene of C2-CDBFV. (**d**) SDS–PAGE analysis of four xylanases. Lane M2: protein molecular mass marker. Lane 1: the strain with empty expression vector without the gene. Lane 2: CDBFV crude enzyme solution. Lane 3: CDBFV purified enzyme solution. Lane 4: C2-CDBFV chimera crude enzyme solution. Lane 5: C2-CDBFV chimera purified enzyme solution. Lane 6: CDBFV-C2 chimera crude enzyme solution. Lane 7: CDBFV-C2 chimera purified enzyme solution. Lane 8: C2-CDBFV-C2 chimera crude enzyme solution. Lane 9: C2-CDBFV-C2 chimera purified enzyme solution.

CDBFV-1 (the target fragment of CDBFV, used to construct chimera C2-CDBFV-C2), C2L (including submodule C2 and linker), LC2 (including linker and submodule C2) (Figure 1a), and CDBFV (used to construct wild-type xylanase), C2-CDBFV (including submodule C2, linker, and CDBFV), and CDBFV-C2 (including CDBFV, linker, and submodule C2) (Figure 1b) constructs appeared on the electrophoresis gel as single bands with estimated molecular masses close to 750 bp (CDBFV-1, C2L, LC2, and CDBFV) and 1500 bp (C2-CDBFV and CDBFV-C2) (Figure 1c), which are close to the theoretical values. Transformed cells containing pET-28a-CDBFV, pET-28a-CDBFV-C2, pET-28a-C2-CDBFV, and pET-28a-C2-CDBFV-C2 were induced to express chimeric xylanases after the sequence accuracy of the recombinant plasmids was verified. The foreign proteins appeared on the SDS-PAGE gel as discrete bands at 29.0 kDa (CDBFV), 44.3–66.4 kDa (C2-CDBFV and CDBFV-C2), and 66.4–97.2 kDa (C2-CDBFV-C2) (Figure 1d). These molecular masses are, as expected, equivalent to theoretical values, which include the value of the chimeric enzyme with both His-tagged ends (Table 1).

### 2.2. Enzyme Properties

#### 2.2.1. Effects of the C2 Submodule on Enzyme Activity and Kinetic Characterization

The degradation activities of four xylanases (CDBFV, C2-CDBFV, CDBFV-C2, and C2-CDBFV-C2) on Corncob xylan, Birch xylan, Bagasse xylan, Avicel, and CMC-Na were determined at pH 5.5 and 60.0 °C. The results showed that the four xylanases had the best degradation activity on Corncob xylan and Birch xylan, followed by bagasse xylan, and almost did not degrade Avicel and CMC-Na (Supplementary Materials Table S2). On different substrates, there were no significant differences in the degradation activities of the four xylanases, suggesting that the C2 submodule had no impact on CDBFV substrate specificity. The binding ability of four xylanases to insoluble substrates (insoluble Corncob xylan, Bagasse xylan, Avicel, and CMC-Na) was measured at pH 5.5 and 60.0 °C. The results showed that the four xylanases had no strong binding ability to insoluble substrates. After reacting at pH 5.50 and 60.0 °C for 2.0 h, the protein binding rates were less than 20.0% (Supplementary Materials Figure S1). The addition of a C2 substructure did not improve the binding of CDBFV to insoluble substrates.

Compared to CDBFV, the activities of C2-CDBFV, CDBFV-C2, and C2-CDBFV-C2 against the Corncob xylan were greater by 18.0–28.0%, and the *K*_M_ values were slightly lower (Table 1). These results indicate that the domain C2 submodule is beneficial for increasing the enzymatic activity of CDBFV.

#### 2.2.2. Effects of the C2 Submodule on pH and Temperature Characterization

The optimal pH of the four xylanases was determined within the pH range from 3.0 to 9.0. According to the pH–activity curve (Supplementary Materials Figure S2a), the maximal activity was observed at pH 5.5. Four xylanases displayed high stability in the pH range of 4.0–9.0 for 60.0 min, according to the pH stability curve (Supplementary Materials Figure S2b), and kept roughly 80.0% of their original activity. The results show that the pH characteristics of CDBFV are unaffected by the C2 submodule.

**Figure 2:**
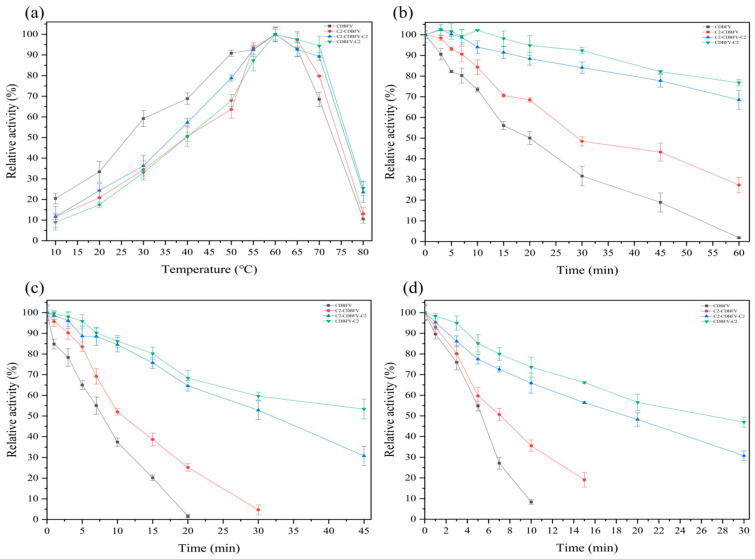
Temperature characterization of four purified xylanases. (**a**) Effect of temperature on enzyme activity: the enzymatic activity was determined at different temperatures of 10.0–80.0 °C and pH 5.5. The enzyme activities of CDBFV, C2-CDBFV, CDBFV-C2, and C2-CDBFV-C2 at pH 5.5 were 1062, 1251, 1260, and 1358 U/mg, respectively, which were defined as 100.0%. (**b**) Temperature stability at 60.0 °C: incubation of the enzymes for 60.0 min at 60.0 °C. (**c**) Temperature stability at 65.0 °C: incubation the enzymes CDBFV-C2 and C2-CDBFV-C2 for 45.0 min, the enzyme C2-CDBFV for 30.0 min, and the enzyme CDBFV for 20.0 min. (**d**) Temperature stability at 70.0 °C: incubation of the enzymes CDBFV-C2 and C2-CDBFV-C2 for 30.0 min, the enzyme C2-CDBFV for 15.0 min, and the enzyme CDBFV for 10.0 min. The enzyme activities of CDBFV, C2-CDBFV, CDBFV-C2, and C2-CDBFV-C2 at pH 5.5 and 60.0 °C were 1062, 1251, 1260, and 1358 U/mg, respectively, in the absence of any treatment, and these values were defined as 100.0%. Relative activity was defined as the percentage of measured high enzyme activity. All values in this research are presented as the mean ± SD of triplicate experiments.

Next, the optimal temperature (100.0 mM, pH 5.5 of sodium citrate buffer) of the four xylanases was determined over a wide temperature range (10.0–80.0 °C). The enzymes were most active at 60.0 °C, as shown in Figure 2a. However, at 70.0 and 80.0 °C, the residual enzyme activity of chimera xylanase C2-CDBFV, CDBFV-C2, and C2-CDBFV-C2 was higher than that of wild-type xylanase CDBFV (Figure 2a). To evaluate thermostability, residual activities of C2-CDBFV, CDBFV-C2, and C2-CDBFV-C2 were measured after incubation at various temperatures (60.0, 65.0, and 70.0 °C) for different times (Figure 2b–d). Both CDBFV-C2 and C2-CDBFV-C2 were stable at the optimal temperature (60.0 °C); the residual activities were 76.8 and 68.5% after 60.0 min treatment. After being treated at 65.0 °C for 30.0 min, the residual activities of the chimeras C2-CDBFV, CDBFV-C2, and C2-CDBFV-C2 were 4.7, 63.6, and 52.8%, respectively. However, the residual activity of wild-type CDBFV was less than 2.0% after being treated at 65.0 °C for 20.0 min (Figure 2c). The residual activity for CDBFV-C2 and C2-CDBFV-C2 was 47.0 and 30.8% after incubation at 70.0 °C for 30.0 min; then, the CDBFV was less than 10.0% at 70.0 °C for 10.0 min (Figure 2d).

Moreover, the half-life times (*t*_1/2_) of four xylanases at different temperatures were compared (Table 1). At 60.0 °C, the *t*_1/2_ of the three chimeras C2-CDBFV, CDBFV-C2, and C2-CDBFV-C2 was 37.5, 122.2, and 93.1 min; it was 1.5, 4.9, and 3.8 times longer than wild-type CDBFV (24.8 min). At 65.0 °C, the *t*_1/2_ of the three chimeras C2-CDBFV, CDBFV-C2, and C2-CDBFV-C2 was 13.7, 44.2, and 31.6 min; it was 1.6, 5.1, and 3.6 times longer than that of CDBFV (8.8 min). At 70.0 °C, the *t*_1/2_ of the three chimeras C2-CDBFV, CDBFV-C2, and C2-CDBFV-C2 was 1.5 times (8.1 min), 4.8 times (25.3 min), and 3.6 times (19.2 min) longer than that of wild-type CDBFV (5.3 min), respectively (Table 1). In summary, these results highlight that the C2 submodule is advantageous for GH11 xylanase CDBFV thermostability, and the C-terminal (CDBFV-C2) connection is preferable to the N-terminal (C2-CDBFV). The chimera CDBFV-C2 has the best heat resistance (Table 1 and Figure 2b–d).

### 2.3. Molecular Modeling and Structural Analysis

**Figure 3:**
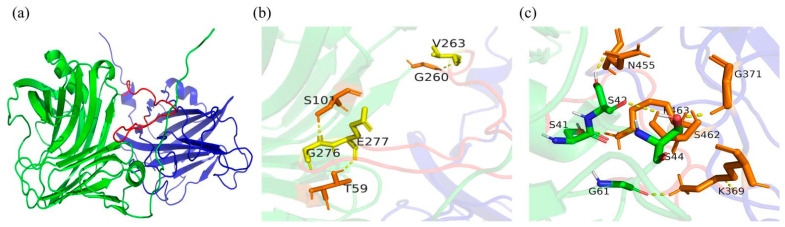
Three-position structure diagram and novel hydrogen bonding interaction of the best chimera CDBFV-C2. The highest-scoring chimera protein structure model was visualized with PyMOL software for visual display, structure superposition, difference analysis, and coloring. Red represents the linker; blue represents the C2; green represents the CDBFV. (**a**) Three-position structure diagram of the best chimera CDBFV-C2. (**b**) Illustration of the newly formed hydrogen bonding interaction between CDBFV and linker in the best chimera CDBFV-C2. (**c**) Illustration of the newly formed hydrogen bonding interaction between CDBFV and C2 in the best chimera CDBFV-C2.

To explore the specific reasons for the difference in the thermal stability of wild-type CDBFV between the N-terminal (C2-CDBFV) and C-terminal (CDBFV-C2) connections of the C2 submodule, the trRosetta server was employed based on the energy-minimized structure algorithm and 15,000 amino acid multiple sequence alignment modeling. Among them, the wild-type CDBFV and C2 submodule exhibit the highest structural consistency with xylanase from *N. patriciarum* (PDB ID: 3WP4) and xylanase 10A of *T. maritima* (PDB ID: 1I8U_A), respectively. The two chimeric xylanases were predicted from the zero-folded protein structure by trRosetta. Five structural models were obtained for each chimera. A modeler was used to fill in the atoms that may be missing in the structure of each obtained model. SAVES V6.0 was used to evaluate all 3D models, and Ramachandran plots of these models were obtained (Supplementary Materials Figure S3). In the best 3D protein models selected, more than 85% of the residue was within the permissible range, indicating that the chimeric structures predicted by trRosetta from the zero-fold protein structure were highly reliable and could be followed up for structural analysis. The obtained model was imported into PyMOL to compare the 3D structure; it was clearly found that there are obvious differences in the structure of chimeric xylanases C2-CDBFV and CDBFV-C2. The protein structure of chimeric xylanase C2-CDBFV is more slender, and the distance between submodule C2 and CDBFV is relatively long (Supplementary Materials Figure S4a). However, the chimeric xylanase CDBFV-C2 is the opposite. The linker is inserted into the β-barrel cavity of CDBFV, and protein interaction can be obviously observed between submodule C2 and CDBFV (Figure 3a).

To further explore the specific interaction of the different connection directions of submodule C2 on the protein structure of CDBFV, we explored the protein structure of the hydrogen bond network between the submodule C2, linker, and CDBFV. First, we analyzed the hydrogen bond interaction between the three parts of the chimeric xylanase C2-CDBFV, and we found that there is no interaction between the submodule C2 and CDBFV. Only two additional hydrogen bonds were formed between the linker and CDBFV, namely, E240-K267 and V244-S260, respectively (Supplementary Materials Figure S4b). Then, we further analyzed the hydrogen bond interaction between the three parts of the chimeric xylanase CDBFV-C2, and we found that all three parts interacted. Three additional hydrogen bonds were formed between the linker and CDBFV, namely, V263-G260, V277-T59, and S101-G276, respectively (Figure 3b). Up to five additional hydrogen bonds were formed between submodule C2 and CDBFV, namely, S41-K463, S42-N455, S44-G371, S42-S462, and G61-K369, respectively (Figure 3c). In brief, by adding submodule C2 and a linker to the N-terminus of CDBFV, the chimeric xylanase C2-CDBFV forms two additional hydrogen bonds compared to wild-type CDBFV. By adding submodule C2 and a linker to the C-terminus of CDBFV, the chimeric xylanase CDBFV-C2 forms eight additional hydrogen bonds compared to wild-type CDBFV. The main reason the thermal stability of the chimera xylanase CDBFV-C2 is better than that of C2-CDBFV is that more new and additional hydrogen bonds are formed.

### 2.4. MD Simulation Details of the Chimeric CDBFV-C2

**Figure 4:**
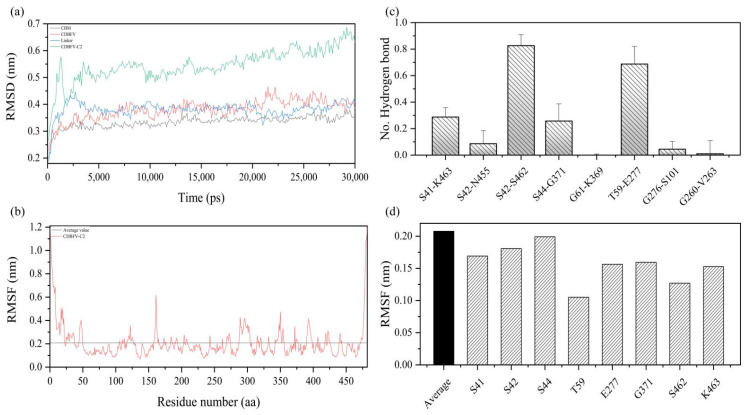
The RMSD, RMSF values, and hydrogen bond stability of the key amino acids of the best chimeric CDBFV-C2. (**a**) RMSD values during a 30.0 ns molecular dynamic simulation of the best chimeric xylanase CDBFV-C2 and its components. (**b**) RMSF values of residues from the best chimeric xylanase CDBFV-C2. The horizontal line represents the average RMSF of all amino acids of the best chimeric xylanase CDBFV-C2. (**c**) The probability of hydrogen bond formation during a 30.0 ns molecular dynamics simulation of the best. chimeric xylanase CDBFV-C2 with eight additional hydrogen bonds. (**d**) RMSD values during a 30.0 ns molecular dynamics simulation of the key amino acid residues of the stable hydrogen bonds.

To explore the protein structural stability of the chimera CDBFV-C2 with the best thermal stability, a 30.0 ns molecular dynamics (MD) simulation was performed on the chimeric xylanase CDBFV-C2 and its components. Generally speaking, the root means square deviation (RMSD) values of the chimeric CDBFV-C2 and its components—CDBFV, Linker, and C2 substructures—are all less than 0.7 nm (Figure 4a). Adding C2 to CDBFV did not cause huge structural differences in C2 and CDBFV parts. In Figure 4a, the structural alterations of C2 and CDBFV were evaluated by RMSD values with black and red lines. It is found that the RMSD values of C2 and CDBFV during 30.0 ns MD simulation were smaller than 0.5 nm, indicating that structures of C2 and CDBFV did not experience huge configurational alteration. This indicates that the protein structure of the chimeric xylanase CDBFV-C2 is relatively stable. Local plasticity of the chimeric xylanase CDBFV-C2 was evaluated by the root mean square fluctuation (RMSF) value of the C_α_ atom, which was calculated using MD simulation data for the last 30.0 ns of the initial structure (Figure 4b). Generally, except for the larger RMSF value of the N- and C-terminal parts, the RMSF value of each amino acid residue of the chimeric xylanase CDBFV-C2 as a whole is less than 0.6 nm, with an average value of 0.2 nm (Figure 4b). The results show that the protein structure of the chimeric xylanase CDBFV-C2 has strong rigidity. In short, the chimeric xylanase CDBFV-C2 has a highly rigid and stable protein structure.

To gain insight into the improved thermostability of the chimeric xylanase CDBFV-C2, the stability of eight additional hydrogen bonds was monitored during the simulation (Figure 4c). During the 30.0 ns MD simulation, hydrogen bonding interactions formed between the amino acid residues S42-S462 and T59-E277 were 82.8 and 62.7 percent, respectively, have a greater than 50.0% probability of forming stable interactions (Figure 4c), while S41-K463 and S44-G371 amino acid residues have a nearly 30.0% probability of forming stable hydrogen bonds. S42-N455, G276-S101, G61-K369, and G260-V263, on the other hand, have a chance of forming stable hydrogen bonds of less than 10.0% (Figure 4c). Even G61-K369 and G260-V263 cannot form stable hydrogen bonds. In addition, the RMSF values of the key amino acid residues of the newly formed four additional hydrogen bonds were counted. The RMSF values of the amino acid residues involved in forming four stable additional hydrogen bonds are all fewer than the average, which can be evaluated in Figure 4d. Analysis of MD simulations reflects that the amino acid residues involved in forming four additional hydrogen bonds improve the stability of the chimeric xylanase CDBFV-C2 protein structure. In short, the improved heat resistance of the chimeric xylanase CDBFV-C2 may be due to the ability of S42-S462, T59-E277, S41-K463, and S44-G371 to form stable additional hydrogen bonds, which is beneficial in increasing the stability of the CDBFV-C2 protein structure and thus significantly improving its thermal stability.

## 3. Discussion

The development of thermostable xylanase is critical for meeting practical industrial demands [5]. To obtain a xylanase chimera with enhanced thermostability, the thermostable domain C2 submodule was first fused to the N-terminus, C-terminus, and both termini of CDBFV in this work. As a result, the chimeric xylanase with the C-terminal C2 submodule has higher thermostability, and fusion at the C-terminus is more effective than fusion at the N-terminus. Through structural analysis, it was determined that the mechanism for the improved thermal stability of xylanase CDBFV-C2 is that S42-S462, T59-E277, S41-K463, and S44-G371 can form stable hydrogen bonds, which were beneficial to increase the stability of the CDBFV-C2 protein structure. Thus, several points are worth discussing.

First, the C2 submodule, in particular, improved the thermostability of xylanase. In the previous study, this effect was investigated by dividing the CBM9_1-2 module into two smaller parts, C1 and C2, which were transplanted into Xyn to create the chimeras Xyn-C1 and Xyn-C2. The Xyn-C2 thermal inactivation half-life (69.3 min) was four or five times longer than that of Xyn (17.6 min), Xyn-C1 (13.4 min), and the original chimera containing CBM9_1-2 (13.8 min) [1,19,20,21]. C2 submodule applied to the N, C, and both sides of CDBFV improved the thermal stability of xylanase by about 1.5, 5.0, and 3.7 times, respectively, compared to wild-type xylanase CDBFV (Figure 2). Previous reports showed that the chimerization of submodule C2 with the N-terminus of *A. niger* xylanase could increase its thermal stability by about four times compared with wild-type xylanase, which is consistent with our conclusions [1]. However, the relationship between CBMs and stability remains controversial. The following three conclusions have been reported: (1) CBMs can improve the thermal stability and optimal temperature of the chimeric enzyme [3,15]; (2) the removal and addition of CBMs have no effect on the thermal stability of the chimeric enzyme [13]; (3) the addition of CBMs reduces the thermal stability of the chimeric enzyme [12,14,16]. The stability of the chimeric enzyme is related not only to its own amino acid sequence but also to the different properties of CBMs. Choosing a CBM derived from a more thermally stable enzyme for chimerization may yield a higher probability of success.

Second, adding CBM to wild enzymes can be performed via various methods. The possibilities include (1) adding CBM to the N-terminus of the wild enzyme [3,12,22]; (2) adding CBM to the C-terminus of the wild enzyme [1,16]; (3) adding CBM to both sides of the wild enzyme [13,15]; (4) adding multiple CBMs to one side of the wild enzyme [23]; (5) replacing or deleting the CBM in the wild enzyme [16]. These results show that different methods of adding CBM have different effects on wild enzymes. However, we cannot predict which way of adding is best in advance. In this work, we found that the C-terminal chimeric module C2 submodule of CDBFV improves thermal stability considerably more than the N-terminal. The thermostable recombinant expands the opportunities for modifying the C-terminal region of GH11 xylanases. Then, the thermal stability of the N-terminal chimeric module C2 sub-module is not significantly improved (Figure 2). The CDBFV’s N-terminal structure has a longer “tail,” which causes the xylanase CDBFV and the module C2 sub-module to be further apart in space [24]. The structure has little influence on each other. The chimeric xylanase C2-CDBFV is also more slender, and the distance between submodule C2 and CDBFV is relatively long, according to our modeling structure (Figure 3a). Simultaneously, the fourth amino acid residue (Cys) at the N end of the CDBFV forms a pair of disulfide bonds with the 172nd amino acid residue (Cys) [24], aiding in the stabilization of the CDBFV’s N-terminal structure. In summary, it is impossible to determine which of the wild enzyme proteins is most suitable for adding CBM, but it can be determined that the side with the most impact on the structure of the wild enzyme protein will produce better outcomes.

Third, another exciting aspect of this study is that fusing the thermostable module C2 submodule to the N-terminus, C-terminus, and both termini of CDBFV increased the activity of CDBFV. Compared with wild-type CDBFV, the specific activities of the chimeric xylanase added to the N-, C-, and both termini of CDBFV increased by 17.8, 18.7, and 27.9%, respectively (Table 1). The same conclusion was reported in a study of chimeras between *A. niger* xylanase and the module C2 submodule [1]. In addition, we also found that the N-terminal (C2-CDBFV) and C-terminal (CDBFV-C2) have no significant difference regarding the improvement of enzyme activity. There is no positional effect, such as thermal stability (Table 1). One possible reason for this is that the two residues Trp175 and Trp71 in the module C2 submodule accomplish a “hand” function [25]. This allows the chimeric enzyme to “unaffected by position, actively grab more substrates” instead of randomly colliding with substrates [1,25]. The enzyme activity of xylanase C2-CDBFV-C2 is the highest among the three chimeras, which may be due to the cumulative effect of the C2 submodule on the increase in enzyme activity [1]. Unfortunately, we did not find that chimeric enzymes improve the efficiency of insoluble substrate hydrolysis (Supplementary Material Figure S1) and substrate specificity (Supplementary Material Table S2). This is different from the result that the C2 submodule and *A. niger* xylanase can improve the binding efficiency of insoluble substrates [1]. The specific reasons may be: (1) the structural similarity between xylanase CDBFV and *A. niger* xylanase is only 38.0%, and their structures and properties are very different, leading to differences between the two results [25,26]; (2) the C2 submodule belongs to the C-type CBM. It only binds to monosaccharides, oligosaccharides, or polysaccharide terminal glycosyls, without grooves, and the binding sites are relatively concentrated, which are easy to embed in the space structure and cannot be completely exposed [25].

Finally, the linker peptide provides the necessary space for each domain to form an active conformation, and the appropriate linker is an important factor that affects the folding of the domains [17,27,28,29,30]. The designed linker peptide will interfere with the thermostability [27,28,29] and enzymatic activity [17,27] of adjacent domains. As previously reported, the flexibility of the linker region is of great significance for enzyme activity [30,31,32], so we used a natural 22-residue linker peptide (PEVLPPLPKESRISEGEAVVVG) to connect these domains. The linker we selected contains four Pro and two Ser residues, which can form flexible hinge regions and aid in creating an active spatial conformation between different domains [1,25]. This may be one of the important reasons why the C2 submodule helps to improve the thermostability and activity of xylanase XynCDBFV.

## 4. Materials and Methods

### 4.1. Bacterial Strains, Vectors, and Reagents

The CDBFV gene from *N. patriciarum* in GenBank (GenBank accession no. KP691331), which contains the 675-base-pair CDBFV sequence, was inserted into pMD19-T [18]. The CBM9_1-2 submodule C2 (residues 871–1059) and its 22-residue linker-peptide (residues 692–713) from *T. maritima* GH10 xylanase A (GenBank accession no. GQ169711) were synthesized into pMD19-T (pMD19-T-LC2L) to form a template for amplification [1,16]. The In-Fusion^®^ HD Cloning Kit, Taq DNA polymerase, restriction endonucleases (*Eco*R I, *Not* I), dNTP, IPTG, DNA marker, and protein marker were purchased from Takara Inc. (Dalian, China). The plasmid miniprep kit and DNA gel extraction kit were obtained from Omega (Taipei, China). A Bradford protein assay kit, pET-28a (+) expression vector, *E. coli* DH5α for cloning, and *E. coli* BL21 (DE3) cells for expression were purchased from TransGen (Beijing, China). All other chemicals were of analytical grade and commercially available.

### 4.2. Construction, Expression, and Purification of the Chimeric CDBFV Variants

To obtain the chimeras, first, a variant with a C2 submodule fused at both termini of CDBFV was constructed. DNA fragments CDBFV-1, C2-linker (C2L), and linker-C2 (LC2) were amplified separately using 0.3 μL pMD19-T-CDBFV or pMD19-T-LC2L as the template (50.0 ng/μL) and P1/P2, P3/P4, or P5/P6 (Supplementary Materials Table S1) as primers (each 2.0 μL) (10.0 μM) with polymerase mixtures (1.0 μL Taq DNA polymerase (5.0 U/μL), 4.0 μL dNTPs (2.5 mM) and 5.0 μL 10× buffer) through 35.0 cycles of denaturation (45.0 s at 95.0 °C), annealing (45.0 s at 55.0 °C), and extension (120.0 s at 72.0 °C). After gel separation, the genes were recovered using a DNA purification kit. DNA fragments CDBFV-1, C2L, and LC2 were ligated sequentially into the expression vector pET-28a (+), which was digested with *Eco*R I and *Not* I. This fusion was performed using an In-Fusion^®^ HD Cloning Kit, as shown in Figure 1a. Then, DNA fragments CDBFV, C2-CDBFV, and CDBFV-C2 were amplified separately using fusion chimera (C2-CDBFV-C2) as the template and P7/P8, P1/P7, or P6/P8 (Supplementary Materials Table S1) as primers. The same method was used to construct two chimeric CDBFV variants and the wild-type CDBFV heterologous expression vector (Figure 1b). *E. coli* DH5α competent cells were then transformed with the recombinant plasmids pET-28a-CDBFV, pET-28a-CDBFV-C2, pET-28a-C2-CDBFV, and pET-28a-C2-CDBFV-C2. Recombinant DNA from the clones, after confirmation by sequencing, was used to transform *E. coli* BL21.

### 4.3. Expression and Purification of the Chimeric CDBFV Variants

Transformed cells were grown in LB medium at 37.0 °C until the OD_600 nm_ reached ~0.7, and xylanases were induced by adding IPTG to a final concentration of 0.5 mM. After further incubation for 15.0 h at 20.0 °C, supernatants were harvested after the cells were disrupted by high-pressure cell breakage (Constant Systems 8TANT, Daventry, Northants, UK) and centrifugation at 13,000 rpm/min for 20.0 min at 4.0 °C. After removing cell debris, both the N-terminal and C-terminal His-tagged enzymes containing the supernatant were purified by affinity chromatography using a Ni-NTA agarose column. Afterward, the purified xylanases were detected using 12.0% polyacrylamide SDS–PAGE analysis, and the enzyme concentration was measured by a Bradford protein assay kit. The gene fragment size and protein molecular mass of the chimeras were calculated by DNAMAN v6 [1,16,18].

### 4.4. Assay Properties of the Chimeric CDBFV Variants

Xylanases’ standard activity was measured using the 3,5-dinitrosalicylic acid (DNS) method [3]. The amount of enzyme consumed by producing 1.0 μmol of xylose per min at 60.0 °C and pH 5.5 (100.0 mM, citrate phosphate buffer) was defined as one unit of xylanase activity. To measure xylanase activity, the reaction system was configured as follows: 450.0 μL of citrate phosphate buffer (100.0 mM, pH 5.5) was mixed with 450.0 μL of the substrate stock solution (1.0% m/v) of Corncob xylan (Yuanye, Shanghai, China) and 100 μL of diluted enzyme solution. After reacting for 10.0 min at 60.0 °C, 1500.0 μL of DNS was added to terminate the reaction, and the reaction mixture was then boiled in a water bath for 5.0 min. After the reaction solution was cooled to room temperature, a SpectraMax (Bio–Rad, Hercules, CA, USA) in a 540.0 nm reader was used to measure the absorbance of the solution supernatant. The blank group reaction without enzyme was performed similarly to the negative control. All measurements were conducted in triplicate.

The enzymatic activity of CDBFV was assayed in parallel with the variants C2-CDBFV, CDBFV-C2, and C2-CDBFV-C2 for assessment of properties. The optimal pH for the purified xylanases activity was determined with buffer pH values from 3.0 to 9.0 at 37.0 °C. The enzymatic stabilities in various pH ranges were judged by measuring the residual enzymatic activities after incubating the enzymatic solution at different buffer pH values from 3.0 to 11.0 at 37.0 °C for 60.0 min. The pH ranges of the buffers were as follows: citrate phosphate buffer (3.0–8.0), Tris-HCl (7.0–9.0), and glycine-NaOH (9.0–12.0). The optimal temperature of purified xylanases was measured within the range from 10.0 to 80.0 °C at pH 5.5. Thermostability was determined at pH 5.5 and 60.0 °C after preincubating the enzyme for different times at 60.0, 65.0, or 70.0 °C. Xylanase-specific activity was assayed on sodium carboxymethyl cellulose (CMC-Na), Avicel, Beechwood xylan, Bagasse xylan, and Corncob xylan (Yuanye, Shanghai, China). The binding ability of the insoluble substrates was analyzed by measuring the protein binding rate in the reaction system after the enzyme solution was reacted at 60.0 °C for 2.0 h [33]. The *K*_M_ and *V*_max_ values for purified xylanases were determined using 0.0%-1.0% Corncob xylan as the substrate in pH 5.5 citrate phosphate buffer at 60.0 °C. The data were fit based on the classic Michaelis–Menton function.

### 4.5. Molecular Modeling and Structural Analysis

The crystal structures of CDBFV from *N. patriciarum* (PDB ID: 3WP4) [26] and C2 from xylanase 10A of *T. maritima* (PDB ID: 1I8U_A) [25] were used as templates to model the binding modules of CDBFV, C2-CDBFV, CDBFV-C2, and C2-CDBFV-C2. The procedure for constructing the three-dimensional structure for all xylanases was performed via the trRosetta online website (https://yanglab.nankai.edu.cn/trRosetta/, accessed on 14 September 2021) according to the methods described previously [24,34,35]. The obtained models were performed using Modeller to complement possible missing atoms in the structure and selection of the best models via the online site SAVES v6.0 [24,34,35]. The obtained models were compared with the visualization software PyMOL, and the molecular effects in the structure were displayed and analyzed [36,37].

### 4.6. MD Simulation Details of the Chimeric CDBFV-C2

The structure of the chimera xylanase CDBFV-C2 was built by trRosetta server [24,34,35]. The MD simulation system was performed via GROMACS program suite version 4.5.7 and Amber ffff99SB force field [38,39]. Sodium or chloride ions (100.0 mM) were added to the system to neutralize the charge [40]. Normal MD simulations were performed in an isothermal–isobaric ensemble for 30.0 ns, which was solvated with TIP3P waters in an octahedral box [41], and the minimal distance between each protein and edge of the box was set to 0.8 nm [42]. Before the simulation, we performed 1000-step energy minimization and equilibrated for 5 ns in NPT ensemble by restraining all heavy atoms [43].

Hydrogen bonds between amino acid residues in the simulation system were analyzed by using g_hbond in the GROMACS suite. Geometrical criteria, which include donor–acceptor distance (≤0.4 nm) and hydrogen-donor–acceptor angle (≤30.0°), are used to calculate hydrogen bond. For each time frame, if both the donor–acceptor distance and the hydrogen-donor–acceptor angle satisfy the criteria, the number of hydrogen bonds will be counted as 1.0, and 0.0 otherwise. The number of hydrogen bonds was calculated based on the whole 30.0 ns simulation (30,000 frames in total) in the system, and the error bar represents one standard error based on the averaged number of hydrogen bonds every 100.0 ps in each system.

## 5. Conclusions

This research shows that the xylanase from *N. patriciarum* and the N- and C-termini (both termini of which fuse a C2 submodule from a hyperthermophilic CBM9_1-2) can enhance thermostability. Compared with wild-type xylanase, the half-lives of the three chimeras increased by about 1.5, 5.0, and 3.7 times, respectively. Structural analysis showed that the thermal stability of the chimera CDBFV-C2 with the best thermal stability was significantly improved by the formation of four relatively stable additional hydrogen bonds (S42-S462, T59-E277, S41-K463, and S44-G371), which is beneficial to increasing the stability of the CDBFV-C2 protein structure, thereby significantly improving its thermal stability.

## Figures and Tables

**Table 1:** Molecular and catalytic characteristics and thermal stability of the four xylanases.

Xylanase	Number of Residues	Mol. Mass ^a^ (kDa)	*V*_max_^b^ (μmol/min/mg)	*K*_M_^b^ (mg/mL)	*t*_1/2_ (60 °C/ 65 °C/70 °C) ^c^ (min)
CDBFV	224	29.4/24.3	1960 ± 100	8.0 ± 0.7	24.8/8.7/5.3
C2-CDBFV	435	53.0/47.9	2210 ± 80	7.2 ± 0.5	37.5/13.7/8.1
CDBFV-C2	435	53.0/47.9	2220 ± 160	7.1 ± 0.9	122.2/44.2/25.3
C2-CDBFV-C2	646	76.5/71.4	2340 ± 120	6.9 ± 0.7	93.1/31.6/19.2

^a^ Molecular masses with and without both His-tagged ends are separated by slash. ^b^ The Michaelis-Menten parameters for Corncob xylan hydrolysis. The reaction was conducted at pH 5.5 (100.0 mM citrate-phosphate buffer) at 60.0 °C. ^c^ The half-times for xylanase inactivation at the indicated temperatures.

## Data Availability

The data presented in this study are available on request from the corresponding author.

## Supplementary Materials

The following supporting information can be downloaded at: https://www.mdpi.com/article/10.3390/ijms23010463/s1.
