# Supplementary material for: Improving the Thermostability of a Fungal GH11 Xylanase via Fusion of a Submodule (C2) from Hyperthermophilic CBM9_1-2
Source: Int J Mol Sci. 2021 Dec 31;23(1):463. doi: 10.3390/ijms23010463 (PMC8745443; doi:10.3390/ijms23010463)
Supplement: Supplementary file 1 [file ijms-23-00463-s001.zip › ijms-1492276-supplementary.pdf]

Supplementary Figure S1. Insoluble substrate binding ability of four purified xylanases. 0.5 mg/mL of four xylanases were respectively added to 1.0 mg/mL insoluble corncob xylan, bagasse xylan, CMC-Na and Avicel in 50.0 mmol/L phosphate buffer (pH = 5.5). After mixing well at 60.0 ℃ and reaction on a rotary mixer for 2.0 h, the supernatant was collected at 10,000 r/min for 10.0 min: the supernatant contained the unbound protein, and the protein concentration was determined by the BCA method. The protein binding rate was calculated. Data are presented as the mean ± SD and are representative of three independent experiments.

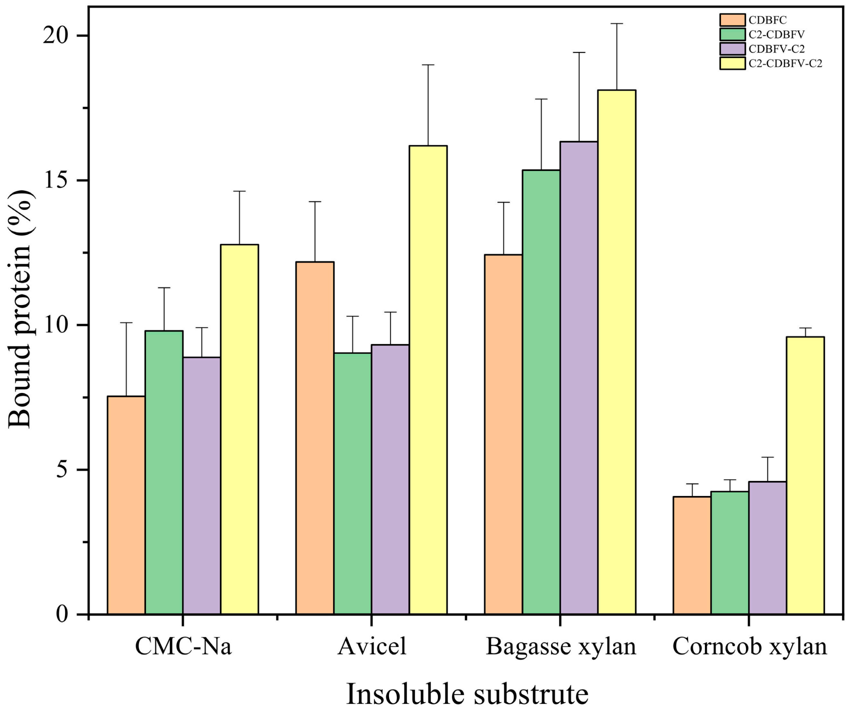

Supplementary Figure S2. pH characterization of four purified xylanases. (a) Effect of pH on enzyme activity; reactions were carried out in different buffers with a concentration of 100.0 mmol/L and a pH range from 3.0-9.0 (McIlvaine buffer) at 37.0 ℃. The enzyme activities of CDBFV, C2-CDBFV, CDBFV-C2 and C2-CDBFV-C2 at pH 5.5 were 849, 1000, 1008 and 1087 U/mg, which were defined as

100.0%. (b) pH stability; the enzymes were incubated for 1.0 h in various pH buffers (pH 3.0-11.0) at 37.0 ℃. The enzyme activities of CDBFV, C2-CDBFV, CDBFV-C2 and C2-CDBFV-C2 at pH 5.5 were 1062, 1251, 1260 and 1358 U/mg, which were defined as 100.0%.

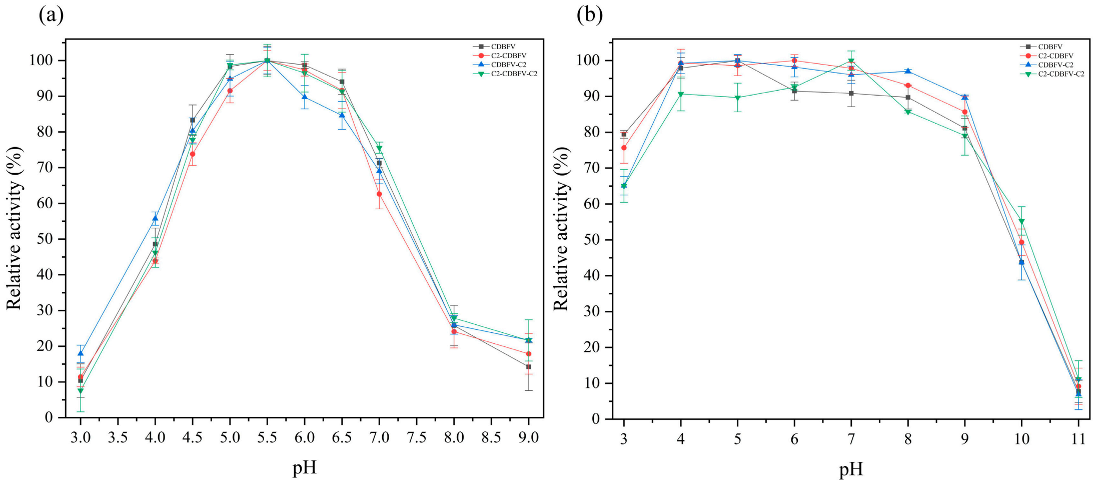

Supplementary Figure S3. Evaluation results of two chimeras. The best model was evaluated by the online website SAVAS for subsequent research. The best 3D model was selected based on the Ramachandran plot value. (a) C2-CDBFV. (b) CDBFV-C2.

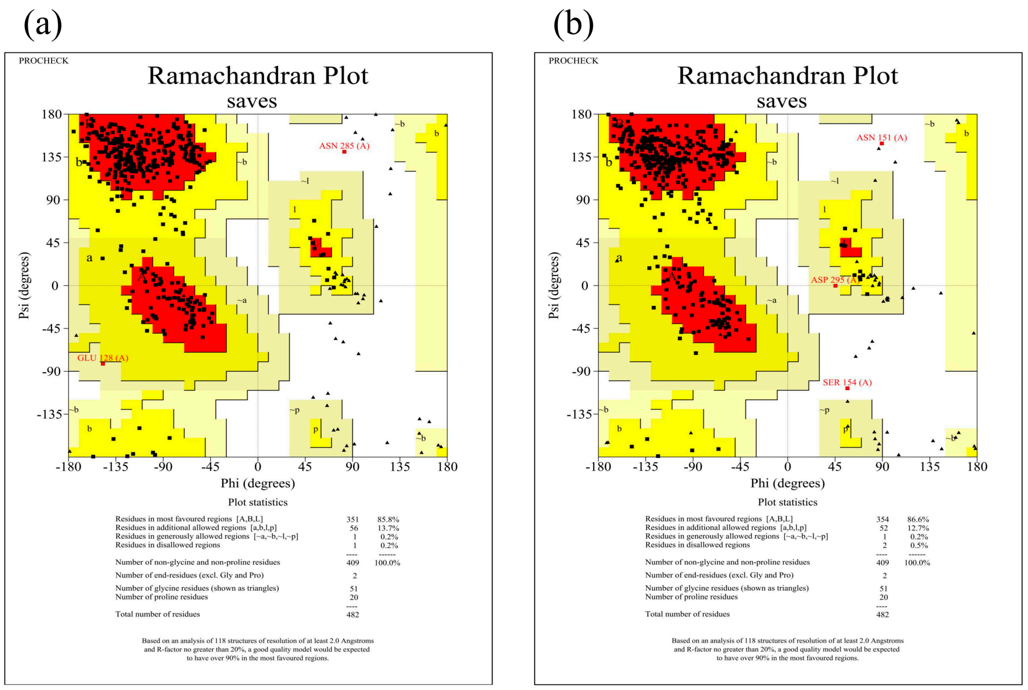

Supplementary Figure S4. Three-position structure diagram and novel hydrogen bonding interaction of the chimera C2-CDBFV. The highest-scoring chimeras protein structure model was visualized with PyMOL software for visual display, structure superposition, difference analysis, and coloring. Red represents the linker; blue represents the C2; green represents the CDBFV. (a) Three-position structure diagram of the chimera C2-CDBFV. (b) Illustration of the newly formed hydrogen bonding interaction in the C2-CDBFV chimera.

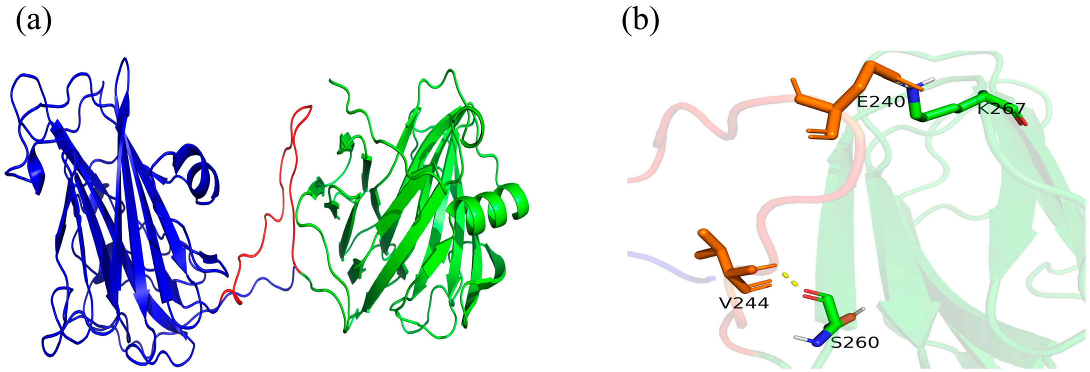

Supplemental Table

Table S1

The primers used in the present research.

Primer	Primer Sequence (5'-3')
P1	AAATGGGTCGCGGATCCGAATTCATGGTAGCGACAGCAAAATACGGAA
P2	GCTGAACTACAGAAACTTTGCCCCACTACCACAGCCTCGCCTTCG
P3	GCGAGGCTGTGGTAGTGGGGCAAAGTTTCTGTAGTTCAGCTTCTC
P4	AGTGGTGGAAGGACCTCAGGACCAATGTAAACCTTTGCGTATGG
P5	ACGCAAAGGTTTACATTGGTCCTGAGGTCCTTCCACCACTTCCAA

P6	TGGTGGTGGTGCTCGAGTGCGGCCGCCTTGATGAGCCTGAGGTTACCGAA
P7	TGGTGGTGGTGCTCGAGTGCGGCCGCACCAATGTAAACCTTTGCGTATGGG
P8	AAATGGGTCGCGGATCCGAATTCCAAAGTTTCTGTAGTTCAGCTTCTC

The *Eco*R-I and *Not*-I site sequences are underlined.

Table S2

Relative activity of chimeric xylanases on different substrates.

Substrate	Relative enzyme activity (%)			
	CDBFV	C2-CDBFV	CDBFV-C2	C2-CDBFV-C2
Corncob Xylan	100±3.64			
Beech Xylan	99.4±4.5	102.4±3.7	98.3±1.3	98.4±0.9
Bagasse Xylan	67.8±4.0	74.0±1.0	73.8±1.5	72.5±2.1
Avicel	0.7±1.4	0.7±4.8	2.4±1.1	2.5±2.5
CMC-Na	0.0±1.5	0.0±1.9	0.0±2.9	0.0±3.9

The enzyme activities of CDBFV, C2-CDBFV, CDBFV-C2 and C2-CDBFV-C2 at pH 5.5 were 1062, 1251, 1260 and 1358 U/mg in the absence of any treatment, which was defined as 100.0%.
